# Fabrication of Luminescent Triple-Cross-Linked Gelatin/Alginate Hydrogels through Freezing-Drying-Swelling and Freezing-Thawing Processes

**DOI:** 10.1021/acs.biomac.4c00289

**Published:** 2024-08-15

**Authors:** Ting-Hsiang Chiu, Shu-Ying Wu, Yi-Chen Yang, Chen-Jie Yan, Yi-Cheun Yeh

**Affiliations:** Institute of Polymer Science and Engineering, National Taiwan University, Taipei 10617, Taiwan

## Abstract

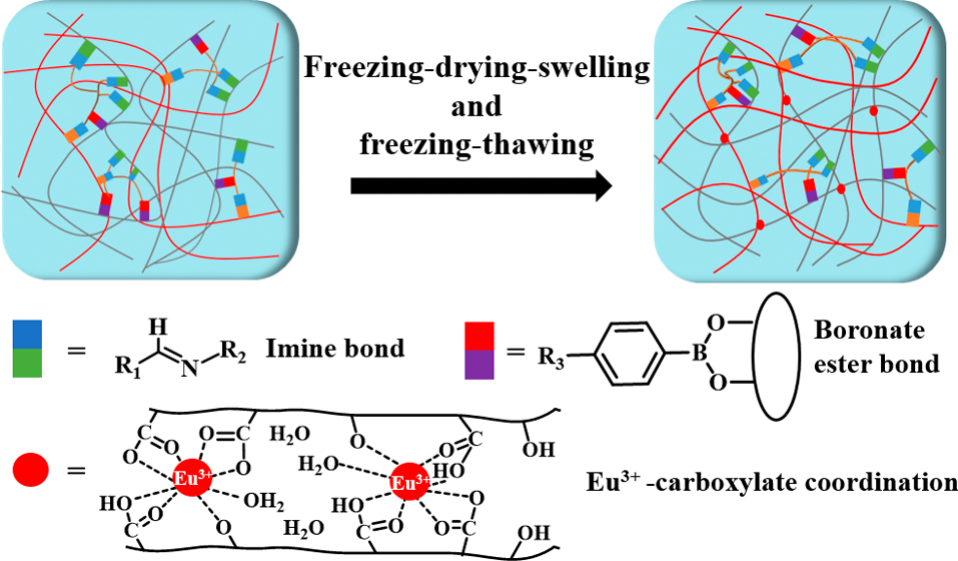

Lanthanide-containing luminescent hydrogels have shown potential for sensing and imaging applications. Nonetheless, integrating lanthanide ions or complexes into the polymer matrix often results in the poor stability and mechanical strength of the hydrogels. This work presents an innovative approach to fabricating luminescent hydrogels with three dynamic cross-links: imine bond, boronate ester bond, and metal–ligand coordination. Europium(III) (Eu^3+^) ions are incorporated into a dual-cross-linked matrix composed of phenylboronic acid-polyethylenimine-modified gelatin (PPG) and alginate dialdehyde (ADA) through a combined treatment involving freeze-drying-swelling (FDS) and freeze–thawing (FT) processes. The FDS process facilitates the formation of additional europium-carboxylate cross-links within the polymeric network to enhance its luminescence and stability, while the FT process strengthens the network physically. The impact of the FDS-FT cycle number on the microstructures and properties of PPG/ADA-Eu^3+^ hydrogels is thoroughly investigated, and their potential for monitoring bacterial growth and detecting copper(II) ions is also demonstrated.

## 1 Introduction

Luminescent hydrogels have garnered significant attention for their outstanding optical characteristics and innovative uses in the controlled delivery of drugs,^1^ biosensing,^2^ three-dimensional (3D) printing,^3^ and tissue engineering.^4^ Luminophores, such as organic dyes,^5^ quantum dots,^6^ and metal complexes,^7^ are commonly utilized as light-emitting sources in the construction of luminescent hydrogels. Recently, luminescent lanthanide complexes have been incorporated into the hydrogel network through noncovalent interaction or covalent connection.^8^ The small size of lanthanide complexes makes their hybridization with hydrogels at the molecular level easy to achieve, facilitating the preparation of a luminescent hydrogel with a uniform structure and stable performance. Lanthanide complexes featuring various functional groups can be synthesized through organic synthesis and coordination assembly techniques, facilitating the integration of these complexes with hydrogels. Given the remarkable luminescent properties of lanthanide complexes (e.g., wide range of luminescence from ultraviolet to near-infrared regions, high color purity, line-like emission, large Stokes shift, and long lifetime), lanthanide-containing hydrogels are more favorable for applications compared to the luminescent hydrogels prepared by incorporating organic chromophores.^8,9^ Most importantly, due to the dynamic coordination of lanthanides, hydrogels containing lanthanide elements typically exhibit adjustable luminescence, responsiveness to stimuli, and self-healing capabilities.^10^

Several methods have been developed to incorporate lanthanide ions or complexes into the polymeric network to form luminescent hydrogels. For example, He et al. fabricated fluorochromic supramolecular hydrogels through europium (Eu)-iminodiacetate coordination, with the iminodiacetate motif modified on the poly(*N,N*-dimethylacrylamide) structure.^11^ The emission of the hydrogels responded to five different stimuli (i.e., pH, heating, metal ions, sonication, and force) due to the reversible hydrogel networks resulting from dynamic metal–ligand coordination. Li et al. reported a luminescent and robust hydrogel using Eu complex-based micelles as the emitting source and cross-linkers to coassemble with exfoliated laponite nanosheets coated with sodium polyacrylate to form supramolecular hydrogel through electrostatic interactions.^5^ The same research group also reported luminescent double-network supramolecular hydrogels, where the first network was poly(vinyl alcohol) (PVA), and the second network was constructed via the copolymerization of acrylamide monomer and the precoordinated allyl-modified 2,6-pyridinedicarboxylic acid lanthanide complex.^12^ Given that the precoordinated complexes work as multifunctional conjunctions to cross-link the polyacrylamide chains, the supramolecular hydrogels performed excellent mechanical properties, stretchability, and self-healing capability. Hu et al. prepared a tough and luminescent alginate/PVA hydrogel with biocompatibility and antibacterial activity using a dual-cross-linking approach of hydrogen bonds and coordination bonds.^13^ In the alginate/PVA network, hydrogen bonds formed between PVA polymers, and lanthanide ions act as physical cross-linkers for alginate by interacting with the carboxylates of alginate. Xie et al. prepared a tough luminescent hybrid hydrogel of PVA and 2,3-pyridinedicarboxylic acid-modified chitosan via frozen-thawing induced cross-linking, and terbium(III) ions were further introduced to the network by forming coordination bonds with the 2,3-pyridinedicarboxylic acid groups.^14^ However, incorporating lanthanide ions or complexes into the polymer matrix generally leads to poor stability and mechanical strength (such as storage modulus less than 10 kPa) of the hydrogels.^15−18^

Here, we hypothesize that through repetitive introduction of lanthanide ions into the hydrogel network via a swelling process, the resulting hydrogels will be incorporated with an optimized amount of lanthanide ions to cross-link polymer chains. These hydrogels can exhibit heightened luminescence and improved mechanical strength, making them more suitable for cutting-edge biomedical applications. Furthermore, hydrogels processed with freezing-thawing are expected to strengthen the hydrogel network owing to the formation of ice crystals, increasing the physical cross-linking in the network.

**Scheme 1:**
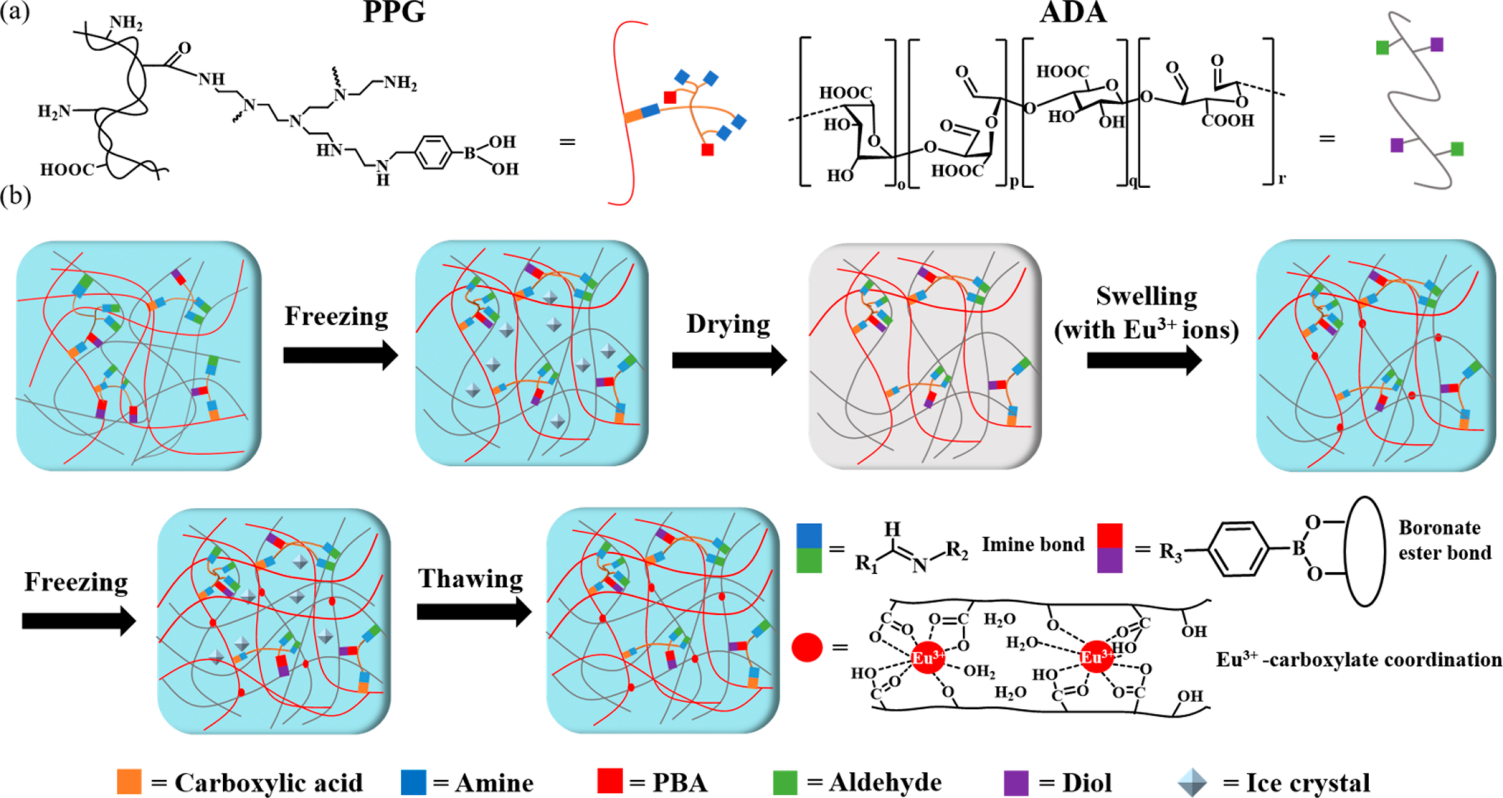
(a) Chemical Structures of PPG and ADA; (b) Schematic Illustrations of PPG/ADA-Eu^3+^ Hydrogel Preparation Process and the Three Cosslinking Mechanisms

In this study, we developed a novel class of luminescent triple-cross-linked hydrogels by integrating the lanthanide ions into the dual-cross-linked polymeric network through the cotreatment of freezing-drying-swelling (FDS) and freezing-thawing (FT) processes (Scheme 1). In our design, phenylboronic acid-grafted polyethylenimine (PBA–PEI)-modified gelatin (PPG) and alginate dialdehyde (ADA) were used to form dual-cross-linked hydrogels through the imine and boronate ester bonds. The Eu^3+^ ions were incorporated into the PPG/ADA hydrogels through FDS process to create additional europium-carboxylate cross-links in the network and enhance luminescent intensity. PPG/ADA-Eu^3+^ hydrogels were further processed with the FT method to increase the mechanical strength of the hydrogels physically. The effect of the FDS-FT cycles (FFC) on the microstructures and properties (i.e., luminescence, rheological behavior, mechanical strength, swelling capacity, and stability) of PPG/ADA-Eu^3+^ hydrogels was systematically investigated. The potential application of PPG/ADA-Eu^3+^ hydrogels in monitoring bacterial growth was also demonstrated.

## 2 Materials and Methods

### 2.1 Materials

Gelatin (from bovine skin, Type B), 1-ethyl-3-(3-dimethylaminopropyl) carbodiimide hydrochloride (99%) (EDC), magnesium(II) chloride (MgCl_2_, 98%), and nitric acid were purchased from Sigma-Aldrich. 4-Formylphenylboronic acid, sodium cyanoborohydride, sodium metaperiodate, diethylene glycol, *N*-hydroxysuccinimide (98%) (NHS), calcium(II) chloride (CaCl_2_, 97%), nickel(II) chloride hexahydrate (NiCl_2_·6H_2_O, 98%), cobalt(II) chloride hexahydrate (CoCl_2_·6H_2_O, 98.0–102.0%), copper(II) chloride dihydrate (CuCl_2_·2H_2_O, >99%), zinc chloride (ZnCl_2_, >98%), and iron(II) chloride tetrahydrate (FeCl_2_·4H_2_O, 98%) were purchased from Alfa Aesar. Sodium alginate and 2-(*N*-morpholino)ethanesulfonic acid (MES) (>99%, <1% water) were purchased from ACROS. Europium(III) nitrate hexahydrate (Eu(NO_3_)_3_·6H_2_O, 99%) was purchased from MorrChem. Phosphate-buffered saline (PBS) buffer, diethyl ether, ammonia, and acetic acid were purchased from JT-BAKER. Dulbecco’s modified eagle medium (DMEM, 4.5 g/L glucose), fetal bovine serum (FBS), and alamarBlue assay were purchased from ThermoFisher.

### 2.2 Preparation of PPG/ADA and PPG/ADA-Eu^3+^ Hydrogels

ADA and PPG were synthesized based on the reported procedure.^19^ The structures of polymers were characterized using nuclear magnetic resonance (NMR, Bruker AVIII HD 400 NMR) and Fourier transform infrared (FT-IR, PerkinElmer Spectrum Two) spectrometers. The molecular weights of polymers were determined using gel permeation chromatography (GPC, Enshine SUPER CO-150). ADA (10 wt %) and PPG (15 wt %) solutions were prepared in PBS solution (pH 7.4). The two solutions were mixed with equal volume, vortexed for one min, and left at room temperature overnight to ensure homogeneity for PPG/ADA hydrogel formation. Freezing-drying-swelling (FDS) and freezing-thawing (FT) processes were further applied to prepare PPG/ADA-Eu^3+^ hydrogel. First, PPG/ADA hydrogel (100 μL) was put into a refrigerator (−80 °C) for 24 h and then lyophilized (−80 °C, 10 mTorr) using a freeze-dryer (UNISS FDM-2) overnight. The lyophilized hydrogel was soaked in the aqueous solution of EuCl_3_·6H_2_O (0.01 mol/L, 5 mL) for 6 h. The swollen hydrogel was frozen again in a refrigerator (−80 °C) for 24 h and thawed at room temperature.

### 2.3 Characterizations of Hydrogels

The morphology of the lyophilized PPG/ADA-Eu^3+^ hydrogels was examined by scanning electron microscopy (SEM, S24800, Hitachi Ltd., Japan), and ImageJ (National Institutes of Health) was used to determine the pore size distribution. The average pore size of each hydrogel was calculated based on 30 different pores and each pore was measured 30 times from different directions.

The chemical compositions of hydrogels were characterized using an attenuated total reflection Fourier transform infrared (FT-IR) spectrometer in the range of 600–4000 cm^–1^ (ATR-FTIR, Nicolet 8700, Thermometer). X-ray photoelectron spectroscopy (XPS, ULVAC PHI 5000 Versa Probe, Japan) measurements were used to analyze the element compositions and chemical state of hydrogels. Inductively coupled plasma mass spectrometry (ICP-MS, PerkinElan DRC II) was used to determine the Eu^3+^ amount in the hydrogel after dissolving the hydrogels in HNO_3_ solution (5 wt %).

### 2.4 Rheological and Mechanical Measurements of Hydrogels

Rheological evaluations of hydrogels were performed using an AR2000EX (TA Instrument) instrument equipped with a 20 mm steel parallel plate, where hydrogels were placed on the Peltier plate. During oscillation strain sweeps, the strain ranged from 0.01 to 1000%, maintained at 1 Hz at 25 °C, with data collected at 10 points per decade.

The mechanical properties of the hydrogels were assessed through compression tests conducted on a materials testing system machine (AGS-X, Shimadzu) with a 10 N load cell at a compression rate of 1 mm/min. The force–displacement information from the samples was transformed into stress–strain graphs, and compressive modulus was calculated by linearly fitting a line through the data points between 10 and 20% strain.

### 2.5 Swelling and Degradation Behaviors of Hydrogels

Hydrogels were weighed (*w*_1_), submerged in PBS buffer at 37 °C, and shaken on a digital rotator at a speed of 100 rpm. The hydrogels were then carefully removed from the solution, excess PBS was removed from the hydrogel surface, and the hydrogels were immediately weighed to determine their weight (*w*_2_). The swelling ratio can be calculated by the following equation
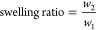
1

For the degradation test, each hydrogel (100 μL) was prepared and frozen over 1 h at −80 °C and then lyophilized. The lyophilized hydrogels (*M*_0_) were immersed in distilled water (1 mL) at 25 °C. At various time intervals (1, 3, 5, and 7 days), the hydrogels were frozen, lyophilized, and reweighted (*M*_d_). The percentage of residual hydrogel mass was calculated according to the following equation
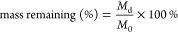
2

### 2.6 Bacteria Culture and Monitoring of Bacterial Growth Experiments

Three distinct bacterial strains, namely *Escherichia coli* (*E. coli*), *Salmonella enterica* (*S. enterica*), and *Staphylococcus aureus* (*S. aureus*), were cultured overnight in tryptic soy broth (TSB) at 37 °C for experimental purposes. The agar disks (diameter = 7 mm, height = 4 mm) formed by TSB solid medium were placed on FFC hydrogels (diameter = 7 mm, height = 6 mm, prepared through the volume of 300 μL). Different strains of bacterial suspension (OD ≅ 0.3, 5 μL) were plated on the agar disc atop the hydrogel, and the samples were incubated at 37 °C to observe the change in luminescence intensity after 4, 8, and 12 h of bacterial incubation. Prior to luminescence detection, FFC3 hydrogels underwent lyophilization to diminish the effect of water on quenching.

### 2.7 Metal Ion Sensing

The FFC3 hydrogels were prepared in a volume of 100 μL, and the lyophilized FFC3 hydrogels were soaked in solutions containing different metal ions (i.e., Ca^2+^, Mg^2+^, Ni^2+^, Co^2+^, Cu^2+^, Zn^2+^, and Fe^2+^) (1 mM, 1 mL) for 1 h at room temperature. The luminescence spectra of the hydrogels were determined by a microplate reader (Synergy H1, BioTek).

### 2.8 Cytocompatibility Study of Hydrogels

The cytocompatibility of hydrogels was evaluated by using the alamarBlue assay with mouse embryonic fibroblasts (MEFs). To prepare hydrogel extracts, hydrogels were sterilized under UV irradiation for 1 h and then immersed in the serum-containing DMEM for 1 day at a volume ratio of 1–10 between hydrogel and media. MEFs were seeded at a density of 1.5 × 10^4^ cells/mL in the 96-well plates and exposed to hydrogel extract solutions in a 5% CO_2_ atmosphere at 37 °C. Cell viability of MEFs was assessed by incubating the media with alamarBlue solution for 4 h. Fluorescence intensity was measured using a microplate reader (Synergy H1, Biotek) at excitation and emission wavelengths of 560 and 590 nm, respectively. Results were normalized to the fluorescence intensity of the hydrogels on day 1, with each experiment repeated three times.

### 2.9 Statistical Analysis

Experiments were conducted in triplicate. Error bars in the figures represent the standard deviation (SD) unless otherwise specified. One-way ANOVA was employed to assess the statistical significance of the differences observed in the data. Significance levels were established at *p* < 0.05, with *, **, and *** denoting *p*-values of <0.05, <0.01, and <0.001, respectively.

## 3 Results and Discussion

### 3.1 Syntheses and Characterizations of Polymers

Alginate dialdehyde (ADA) was synthesized by oxidizing alginate with sodium periodate according to the reported procedure,^20^ and the degree of oxidation of ADA was 22% determined by the hydroxylamine hydrochloride titration method. The structural characterization of ADA was confirmed by nuclear magnetic resonance (NMR) and Fourier transform infrared (FT-IR) spectrometers. Given that the oxidation of alginate leads to the formation of multiple aldehyde species and hemiacetals, the appearance of two new peaks that emerged at around 5.4 and 5.6 ppm in the ^1^H NMR spectra of ADA corresponded to the hemiacetalic protons formed from aldehydes and neighboring hydroxyl groups (see Supporting Information Figure S1a,b). In the FT-IR spectra of ADA, the carbonyl stretching band peak of ADA appeared at 1730 cm^–1^ (see Supporting Information Figure S1c). The molecular weight of ADA was obtained through GPC measurement, showing that the weight average molecular weight (*M*_w_) of ADA was ∼121 kDa with a polydispersity index (PDI) of 1.23 compared to alginate (*M*_w_ = ∼164 kDa, PDI = 1.23) (see Supporting Information Figure S2).

Phenylboronic acid-polyethyleneimine-modified gelatin (PPG) was synthesized by grafting phenylboronic acid-polyethyleneimine (PBA–PEI) to gelatin through amide coupling.^20^ In the NMR spectrum of PBA–PEI, the peaks at 7.2 and 7.5 ppm corresponded to the protons on the phenyl rings of phenylboronic acid, and the peaks in the range of 2–3 ppm corresponded to the protons on the main chain of PEI. In the NMR spectrum of PPG, the peaks at 7.3 and 7.6 ppm corresponded to the protons on the phenyl rings of phenylboronic acid, despite hindrance by aromatic residues in gelatin at 7.2 ppm. Additionally, the peaks at 2–3 ppm corresponded to the protons from the main chain of PEI (Supporting Information Figure S3a–c). In the FT-IR spectrum of gelatin, the O–H stretching vibration, C=O stretching vibration, and N–H bending vibration in gelatin appeared at 3414, 1639, and 1540 cm^–1^, respectively (see Supporting Information Figure S3d). In the FT-IR spectrum of PPG, the C–N bond at 1475 cm^–1^ was shown, along with the C=O peak of gelatin at 1643 cm^–1^, which was shifted from 1639 cm^–1^. Both NMR and FT-IR analyses demonstrated that PBA–PEI was successfully grafted onto the gelatin. The *M*_w_ of gelatin and PPG were ∼72 kDa (PDI = 1.25) and ∼128 kDa (PDI = 1.28), respectively (see Supporting Information Figure S4).

### 3.2 Formations and Characterizations of Hydrogels

The PPG/ADA hydrogels were obtained by forming imine and boronate ester bonds in the polymeric network after mixing the PPG and ADA solutions and staying overnight. The amines of PPG and aldehydes of ADA formed imine bonds, and the phenylboronic acids of PPG and the diols of ADA formed boronate ester bonds. The abundance of carboxyl groups in the PPG/ADA hydrogels serves as adsorption sites for Eu^3+^ ions by forming europium-carboxylate coordination in the network. The Eu^3+^ ions were incorporated into the PPG/ADA network through freezing-drying-swelling (FDS) processes, forming triple-cross-linked PPG/ADA-Eu^3+^ hydrogels. Furthermore, PPG/ADA-Eu^3+^ hydrogels went through a freezing-thawing (FT) process to strengthen the structure physically. When the PPG/ADA-Eu^3+^ hydrogels were frozen, water transformed into ice crystals. This transformation caused the ice crystals to push the PPG/ADA network and Eu^3+^ ions out, confining them within the spaces between adjacent ice crystals.^21^ Consequently, the hydrogel separates into two distinct phases upon freezing: one consists of ice, and the other consists of polymers and ions. In this polymer-ion phase, the distance between Eu^3+^ ions and the PPG/ADA network was significantly reduced compared to that of the original hydrogel structure, leading to a substantial increase in the concentration of both components as well as facilitating the coordination bond formation between the PPG/ADA polymers and Eu^3+^ ions. As a result of the freeze–thaw (FT) process, the initially loose network transformed into a densely cross-linked structure, thereby improving the mechanical properties of hydrogels.

The numbers of FDS and FT processes played a critical role in determining the microstructures and properties of the PPG/ADA-Eu^3+^ hydrogels as the introduction of Eu^3+^ ions through the FDS process can chemically generate more coordination cross-links in the network, and the FT process can physically increase the strength of the network. Here, different numbers of FDS-FT cycles (FFC) were used in preparing the PPG/ADA-Eu^3+^ hydrogels, forming in the hydrogels of FFC1, FFC2, and FFC3 representing the numbers of FFC for 1, 2, and 3, respectively. The PPG/ADA hydrogel without the treatment of FFC was named FFC0 in this study.

Inductively coupled plasma mass spectrometry (ICP-MS) was used to determine the Eu^3+^ amount of the PPG/ADA-Eu^3+^ hydrogels, showing that the Eu^3+^ amount for FFC1, FFC2, and FFC3 hydrogels was 0.42, 0.60, and 0.64 mmol/g, respectively. The increase in the amount of Eu^3+^ in the hydrogels with a higher FFC was mainly attributed to the driving force of the FDS cycles to result in sufficient Eu^3+^ ions in the carboxylate chelating sites of PPG/ADA-Eu^3+^ hydrogels.

**Figure 1:**
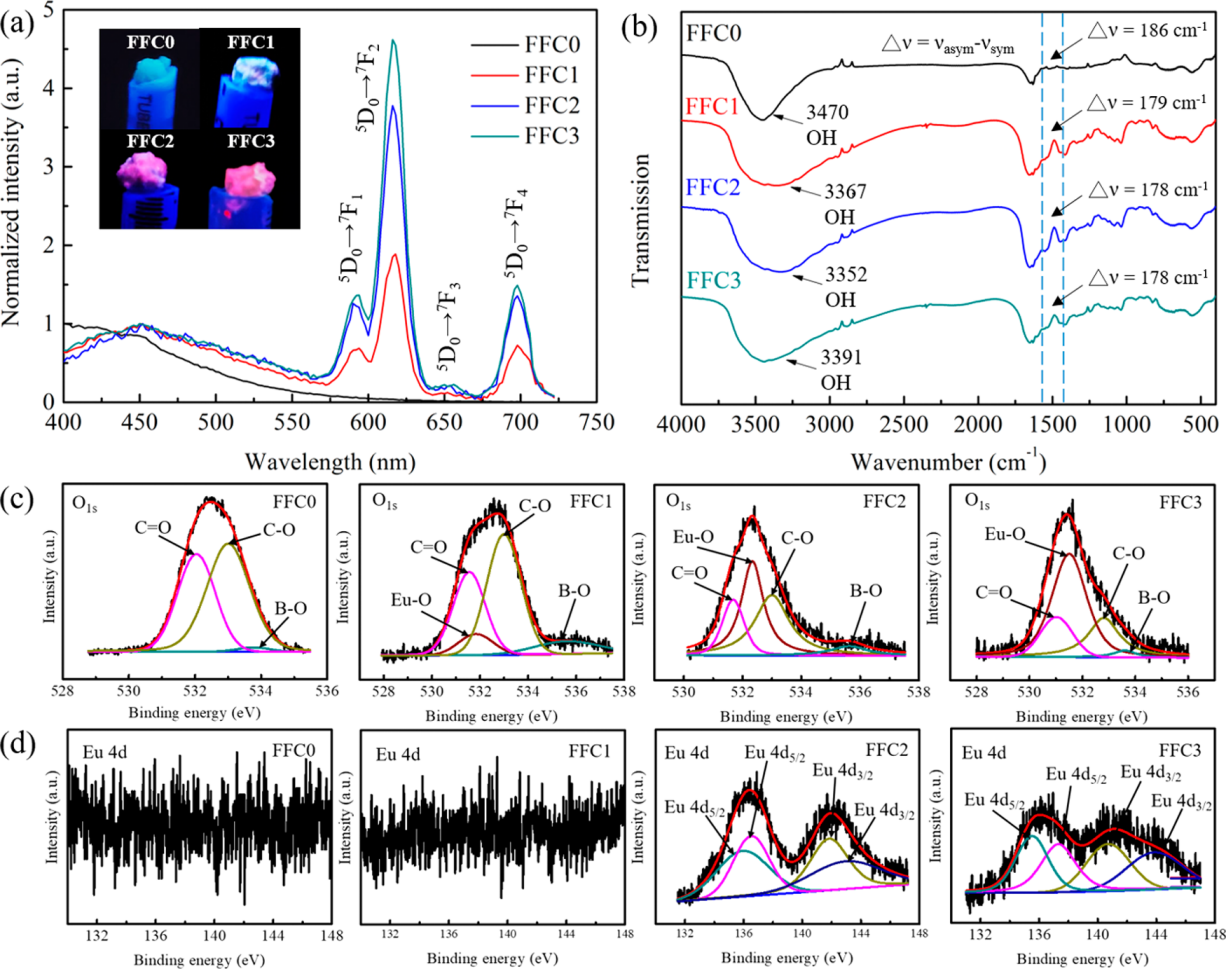
(a) Luminescence images and spectra of the hydrogels. (b) FT-IR spectra of the hydrogels. XPS (c) O 1s and (d) Eu 4d spectra of the hydrogels.

In the luminescent spectra of PPG/ADA-Eu^3+^ hydrogels, a series of typical peaks corresponding to the intra ^5^D_0_ → ^7^F_0–4_ transitions was revealed under the excitation at 300 nm^22^ (see Figure 1a). The luminescence intensity of the PPG/ADA-Eu^3+^ hydrogels was in a Eu^3+^ amount dependent manner upon the increasing of FFC during the hydrogel preparation. The luminescence intensity ratios of peak (IRP) *I*_616_/*I*_592_ were used to evaluate the luminescence property of PPG/ADA-Eu^3+^ hydrogels, where a greater IRP value indicated better red color purity. The IRP values of FFC1, FFC2, and FFC3 hydrogels were 2.7, 3.4, and 3.5, respectively. The higher IRP value of the FFC3 hydrogel could be due to the significant amount of the coordinate bonding between Eu^3+^ ions and carboxylate groups of polymer chains to cause the expulsion of some water molecules from their coordination shell and resulted in the enhanced luminescence intensity at 616 nm.^23^ On the other hand, the amount of free Eu^3+^ ions in the hydrogel decreases as the intensity at 616 nm increases,^15^ indicating the IRP value can also be used to confirm that Eu^3+^ has successfully coordinated within the FFC hydrogel network.^13^

FT-IR analysis was also performed to validate the Eu^3+^ coordination in the PPG/ADA-Eu^3+^ hydrogels. The result showed that the vibration intensity of carboxyl groups at 1660 cm^–1^ significantly decreased along with the higher adsorption of Eu^3+^ ions (see Figure 1b), indicating the critical role of the carboxyl groups of polymer chains in the formation of complexes with Eu^3+^ ions. It was also noticed that the peak of the O–H bond at 3470 cm^–1^ shifted to a lower wavenumber of 3367, 3352, and 3391 cm^–1^ due to the interaction between the Eu^3+^ ions and OH groups.^24^ The appearance of new peaks at 1597 and 1420 cm^–1^ was selected for the symmetric stretching vibration and asymmetric stretching vibration of carboxylate groups, and the separation of symmetric stretching vibration and symmetric stretching vibration (Δν) is commonly used to characterize the interaction mode of the carboxylate group.^25^ It is monodentate coordination for the carboxylate group when the Δν is greater than Δν sodium, while it is bidentate coordination when the Δν is less than Δν sodium. The Δν for the sodium/carboxylate group (Δν sodium) in FFC0 hydrogel was about 186 cm^–1^, and the Δν was 179, 178, and 178 cm^–1^ for FFC1, FFC2, and FFC3 hydrogels, respectively. Therefore, the coordination interaction in PPG/ADA-Eu^3+^ hydrogels was bidentate coordination, including chelating and bridging.

The chemical compositions of the PPG/ADA-Eu^3+^ hydrogels were characterized via X-ray photoelectron spectroscopy (XPS) (see Supporting Information Figure S5). In the XPS spectrum of the FFC0 hydrogel, only boron (B), carbon (C), nitrogen (N), and oxygen (O) were detected at 187, 285, 400, and 530 eV, respectively. Once PPG/ADA-Eu^3+^ hydrogels were prepared through FFC, a new peak of Eu was detected at 136 eV. Also, the atomic percentage of Eu in FFC3 hydrogel (4.61%) was much higher than that in FFC2 (2.04%) and FFC1 (0.14%) hydrogels. The typical high-resolution spectra of the O 1s regions of FFC0-FFC3 hydrogels are shown in Figure 1c. With respect to the deconvoluted O 1s spectra of the FFC0, three curve-fitted peaks at binding energies of 531, 532, and 534 eV were identified as corresponding to the C=O, C–O, and B–O bonds, respectively, indicating the varied chemical states of oxygen atoms within the hydrogel.^26^ In contrast, the deconvoluted O 1s spectra for FFC1, FFC2, and FFC3 hydrogels revealed four distinct peaks, showing a decrease in the relative content of oxygen atoms associated with C–O and C=O bonds. This reduction is attributed to the partial consumption of C–O and C=O bonds in forming coordination bonds with Eu^3+^ ions, as evidenced by the emergence of new curve-fitted peaks at 532 eV indicative of O–Eu bonds.^26^

The Eu signals in XPS spectra became noticeable in the FFC2 and FFC3 hydrogels (see Figure 1d). In the XPS spectrum of FFC3, Eu 4d orbital was detected at the characteristic binding energy of 136 eV, doublet peaks at the binding energy of 136 and 141 eV assigned to Eu 4d_5/2_ and Eu 4d_3/2_ with a spin–orbit splitting of 5 eV were observed, respectively, which could be attributed to the multiplet structure of the trivalent 4d4f configurations.^27^ Moreover, the satellite lines on the high binding energy side of the Eu 4d levels (137 and 144 eV for 4d_5/2_ and 4d_3/2_, respectively) were observed, which might be ascribed to the O(2p) → Ln(4f) charge-transfer excitation, suggesting the formation of a Eu–O bond.

### 3.3 Microstructures of Hydrogels

**Figure 2:**
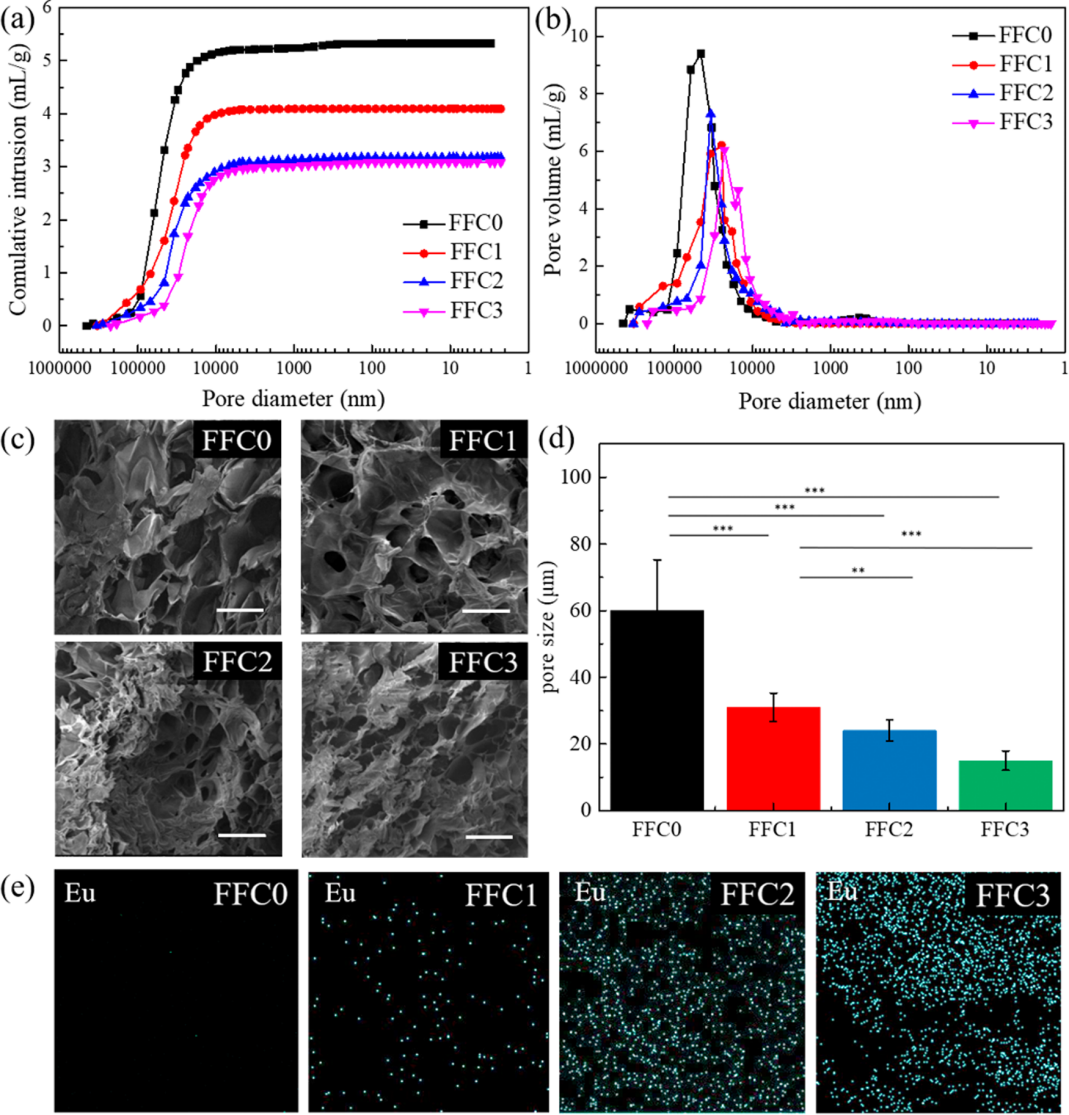
Pore size distributions were presented as (a) cumulative pore volume and as (b) differential pore volume (d*V*/d*D*) of lyophilized hydrogels. (c) Representative SEM images of the lyophilized hydrogels. Scale bar = 50 μm. (d) The average pore size of hydrogels determined by SEM images. Significance was set at *p* < 0.05, with ** and *** denoting *p*-values of <0.01 and <0.001, respectively. (e) SEM elemental distribution analyses of lyophilized hydrogels on Eu.

Mercury intrusion porosimetry (MIP) was applied to determine the porosity of the hydrogels quantitatively (Figure 2a,b and Table 1). In the total intrusion volume, FFC0 hydrogel presented the highest value (5.95 mL/g), followed by FFC1 (5.83 mL/g), FFC2 (5.48 mL/g), and FFC3 (4.81 mL/g) hydrogels. The median pore diameters of FFC0, FFC1, FFC2, and FFC3 hydrogels were 64.34, 47.55, 39.05, and 28.51 μm, respectively. In addition, the FFC3 hydrogel has the highest total pore area (2.79 m^2^/g), followed by FFC2 (2.18 m^2^/g), FFC1 (0.83 m^2^/g), and FFC0 (0.55 m^2^/g) hydrogels. All of the hydrogels presented porosities in the range of 84–89%. Hence, with an increase in the number of FFC, the cross-linking density of the PPG/ADA-Eu^3+^ hydrogels also increased, leading to a more compact pore structure and a reduction in porosity. These findings indicated that the incorporation of Eu^3+^ ions enhanced the structural integrity of the hydrogel through the formation of chemical coordination cross-links. Moreover, during the freezing process, the formation of ice crystals within the amorphous regions of the hydrogel promoted the development of polymer crystallites, acting as physical cross-links between the polymer chains of the hydrogel. Consequently, more FFC resulted in a more compact hydrogel network structure.

**Table 1:** Microstructural Analyses of Hydrogels Using Mercury Intrusion Porosimetry

	total intrusion volume (mL/g)	median pore diameters (μm)	total pore area (m^2^/g)	porosity (%)
FFC0	5.32	53.65	1.45	85.16
FFC1	4.09	44.77	0.40	83.59
FFC2	3.17	36.04	2.40	88.64
FFC3	3.08	24.34	1.68	86.24

We processed the hydrogel preparation in solutions containing different Eu^3+^ concentrations with FFC = 3 to investigate the incorporated Eu^3+^ amounts and the microstructures of hydrogels. The results showed that the Eu^3+^ amounts for hydrogels processed in the Eu(NO_3_)_3_ solutions with concentrations of 0.005 and 0.015 M with FFC = 3 were 0.56 and 0.79 mmol/g, respectively, and the FFC3 hydrogel processed in the Eu(NO_3_)_3_ solutions with concentrations of 0.010 M with FFC = 3 was 0.64 mmol/g. The median pore diameters of the hydrogels processed in the Eu(NO_3_)_3_ solutions with concentrations of 0.005 and 0.015 M with FFC = 3 were 42.49 and 8.11 μm **(**see Supporting Information Figure S6 and Table S1), respectively, and the FFC3 hydrogel was 24.34 μm. Therefore, with the same FFC, the initial concentration of Eu(NO_3_)_3_ solution used during the process was also related to the final Eu^3+^ amount in the hydrogels, affecting the porous structures of the hydrogels. A denser network was observed by processing the PPG/ADA-Eu^3+^ hydrogel preparation with the solution of a higher Eu^3+^ concentration.

Scanning electron microscopy (SEM) was further used to reveal the porous microstructures of lyophilized hydrogels (see Figure 2c), and the average pore sizes of FFC0, FFC1, FFC2, and FFC3 hydrogels were 60.0 ± 15.2, 32.0 ± 4.7, 25.0 ± 3.2, and 15.4 ± 2.9 μm, respectively (see Figure 2d). Elemental distribution analysis was also performed to reveal the elemental distribution of the hydrogels. Several elements (i.e., C, O, N, and B) were observed in these FFC0-FFC3 samples due to the PPG/ADA polymeric network (see Supporting Information Figure S7). It was also noticed that the elemental intensity of Eu was increased after a higher number of FFC was applied in preparing hydrogels, confirming the amount of Eu^3+^ ions introduced to the hydrogel network based on the number of FFC (see Figure 2e).

### 3.4 Rheological and Mechanical Properties of Hydrogels

**Figure 3:**
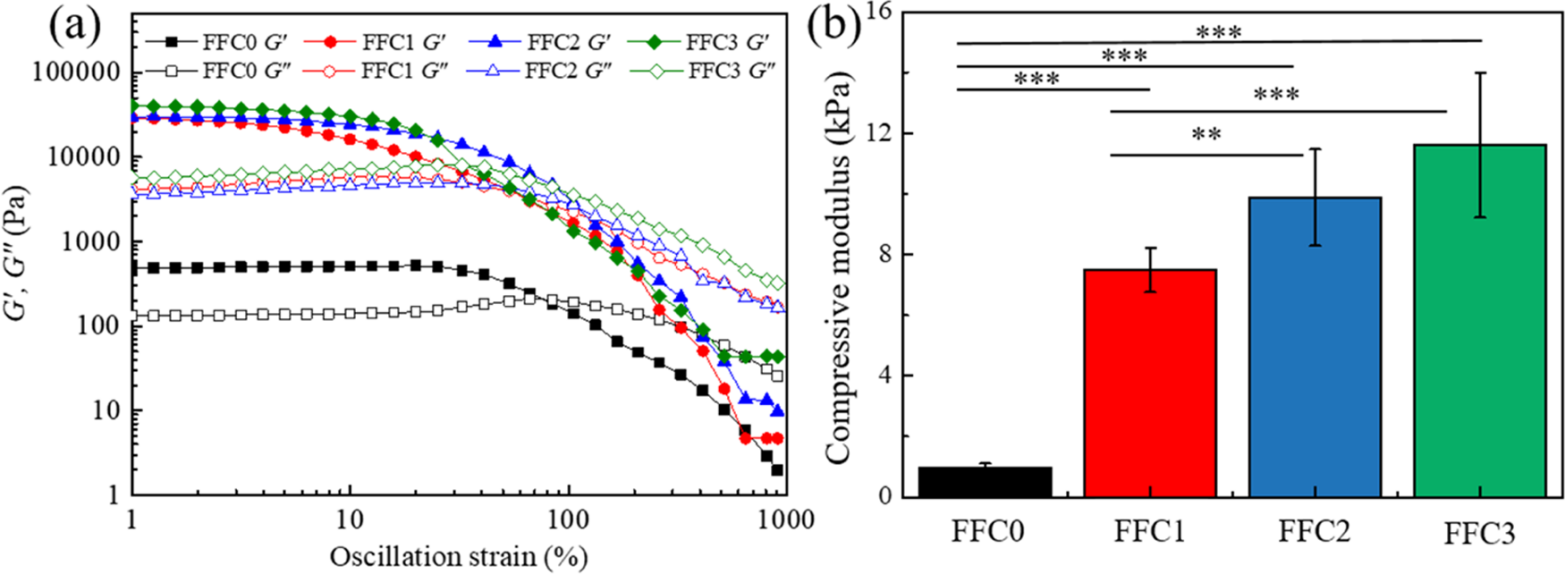
(a) The continuous strain sweeps of hydrogels. (b) The compressive modulus of FFC hydrogels. The significance was set as *p* < 0.05 with ** or *** indicating *p* < 0.01 or 0.001, respectively.

The effect of the number of FFC on the rheological properties of the PPG/ADA-Eu^3+^ hydrogels was investigated. In the oscillation strain sweep, the storage moduli (*G′*) of FFC0, FFC1, FFC2, and FFC3 hydrogels were ∼0.5, 25, 26, and 28 kPa, respectively (see Figure 3a and Table 2). The cross-linking density of hydrogels can be further calculated by the following formula^28^
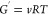
Where *G′* represents storage modulus in Pa, ν represents cross-linking density in mol/m^3^, *R* represents gas constant (8.314 J K^–1^ mol^–1^), and *T* represents the temperature in K. After the calculation, the cross-linking densities of the FFC0, FFC1, FFC2, and FFC3 hydrogels were ∼0.18, 12.50, 12.82, and 15.39 mol/m^3^, respectively. Therefore, the higher number of FFC enhanced the rheological properties and cross-linking densities of PPG/ADA-Eu^3+^ hydrogels.

**Table 2:** Rheological Analyses of the Hydrogels

	storage modulus (kPa)	loss modulus (kPa)	cross-linking density (mol/m^3^)	flow point (%)
FFC0	0.45 ± 0.01	0.15 ± 0.05	0.18 ± 0.01	84.34 ± 1.31
FFC1	30.97 ± 0.38	3.03 ± 0.12	12.5 ± 0.07	46.25 ± 2.30
FFC2	31.76 ± 0.34	4.01 ± 141.06	12.82 ± 0.21	41.01 ± 0.15
FFC3	38.13 ± 0.38	7.53 ± 0.13	15.39 ± 0.41	32.52 ± 0.08

Besides, the flow points of the FFC0, FFC1, FFC2, and FFC3 hydrogels were ∼84.34, 46.25, 41.01, and 32.52%, respectively. The flow point of FFC3 hydrogel was lower than that of FFC0 hydrogel, which might be attributed to the structural changes that occurred during the freeze–thaw process, including the crystallization and melting of water molecules within the hydrogel as well as the destruction and regeneration of the hydrogel structure during freezing. These changes can affect the structure and mechanical properties of the hydrogel, resulting in increased strength but potentially causing a loss of some extensibility.

The compression test was further performed to reveal the stiffness of PPG/ADA-Eu^3+^ hydrogels. The compressive moduli of FFC0, FFC1, FFC2, and FFC3 hydrogels were ∼1, 8, 10, and 12 kPa, respectively (see Figure 3b and Supporting Information Figure S8). As a control, we also investigated the impact of freeze–thaw (FT) cycles on PPG/ADA hydrogels (see Supporting Information Figure S9). There was a significant improvement in the compressive modulus of PPG/ADA hydrogel in the first treatment of the FT cycle, while the compressive moduli of the three samples (FT1, FT2, and FT3) were nearly in the same range (∼5.8 kPa). Therefore, additional FT cycles did not enhance the mechanical performance of PPG/ADA hydrogel in the absence of added Eu^3+^ ions. The elasticity of the FFC3 hydrogel was examined through a compression-uncompression process under strains ranging from 0 to 50% and a stress of 10 N. The FFC3 hydrogel displayed moderate fatigue resistance during five continuous cycles of loading and unloading at 50% strain, showing its elasticity as it fully recovered its original shape (see Supporting Information Figure S10)

These rheological and compression results suggested that introducing Eu^3+^ ions to the PPG/ADA network through FFC improved the strength of the PPG/ADA-Eu^3+^ hydrogels. These mechanical analyses of PPG/ADA-Eu^3+^ hydrogels also agreed with their structural observations as more FFC generated a denser hydrogel network. It was also highlighted that the number of FFC can be used to fine-tune the microstructures and mechanical properties of PPG/ADA-Eu^3+^ hydrogels, providing a simple and straightforward method to customize luminescent lanthanide-containing hydrogels for specialized utility.

### 3.5 Swelling, Degradation, and Self-Healing Tests of PPG/ADA-Eu^3+^ Hydrogels

**Figure 4:**
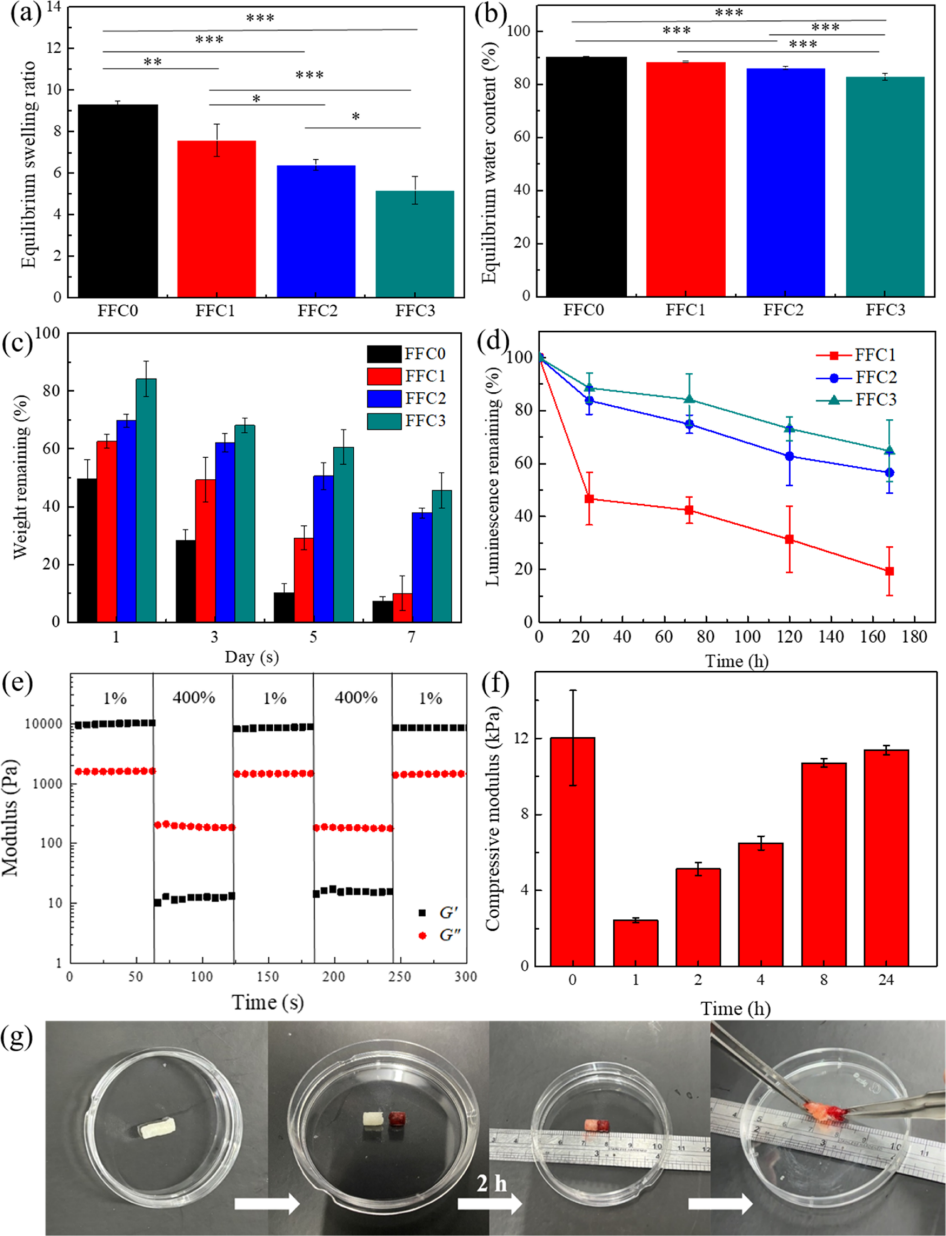
(a) Equilibrium swelling ratio and (b) equilibrium water content of the hydrogels in PBS (pH 7.4). The significance was set as *p* < 0.05 with *, **, or *** indicating *p* < 0.05, 0.01, or 0.001, respectively. (c) Degradation and (d) luminescence remaining of hydrogels. (e) Continuous step strain sweep test of FFC3 hydrogels under alternating high strain (400%) and low strain (1%). (f) Self-healing efficiency tests of FFC3 hydrogels. (g) Demonstrations of the self-healable FFC3 hydrogel in macroscale.

The equilibrium swelling behaviors of the PPG/ADA-Eu^3+^ hydrogels were investigated by their capacity to absorb and retain water molecules within the structure. The equilibrium swelling ratio of the hydrogel samples decreased from 9.5 to 4.7 (g/g) when the number of FFC increased from 0 to 3 (see Figure 4a). Furthermore, the water contents of the FFC0, FFC1, FFC2, and FFC3 hydrogels were ∼90.25, 88.71, 86.27, and 83.02%, respectively (Figure 4b). Typically, the swelling behavior and water retention of hydrogels are associated with their internal architecture.^29^ Studies have shown that hydrogels with a looser internal structure tend to swell more rapidly due to their greater porosity, which facilitates the diffusion and exchange of water.^30^ Therefore, the FFC0 hydrogel with a larger pore size exhibited a greater swelling ratio and higher water content than the hydrogels treated with FFC. Furthermore, the swelling properties of the PPG/ADA-Eu^3+^ hydrogels can be adjusted by changing the number of FFC, where the denser structure of the FFC3 hydrogels resulted in lower water retention within the network.

To explore the correlation between the luminescence decrease and hydrogel degradation, the degradation of PPG/ADA-Eu^3+^ hydrogels was evaluated in water by simultaneously recording the weight loss of hydrogels and the amount of decreased luminescence at regular intervals. The FFC0, FFC1, FFC2, and FFC3 hydrogels were degraded ∼93, 90, 71, and 50%, respectively, after the immersion of 7 days in PBS solution (see Figure 4c). Meanwhile, the luminescence of FFC1, FFC2, and FFC3 hydrogels decreased by ∼80, 43, and 35%, respectively (see Figure 4d). The results suggested that the incorporation of FFC in the synthesis of PPG/ADA-Eu^3+^ hydrogels not only enhanced the cross-linking density of the network but also led to slower degradation and improved the luminescence profile of the hydrogels. Moreover, the release of Eu^3+^ ions was primarily associated with the degradation of the hydrogel rather than being governed by diffusion processes. The Eu^3+^ ions, which were coordinated with the carboxyl groups of ADA, began to escape from the network as the hydrogel degraded due to the dissociations of imine and boronate ester bonds. Notably, the degradation profile of the hydrogel was correlated to the reduction in luminescence, indicating that a decrease in luminescence could serve as an indicator of hydrogel degradation.

The presence of dynamic bonds (i.e., imine bonds, boronate ester bonds, and coordination bonds) in the PPG/ADA+Eu^3+^ hydrogels allowed the hydrogel network to be self-healable. Here, the FFC3 hydrogel was used as a representative sample to demonstrate the self-healing behavior of PPG/ADA-Eu^3+^ hydrogels. Rheological results showed that the hydrogel structure can be destroyed into a liquid-like status under high strain (∼400%) while recovering to a gel state under low strain (∼1%) (see Figure 4e). The healed hydrogels had modulus values similar to those of the original FFC3 hydrogels, indicating that FFC3 hydrogels presented an excellent self-healing manner. The self-healing efficiency of FFC3 hydrogels was quantified by determining the compressive modulus changes of the hydrogels during the time course. The self-healing efficiency of the FFC3 hydrogel was ∼20, 43, 54, 89, and 95% after 1, 2, 4, 8, and 24 h, respectively (see Figure 4f). In addition, FFC3 hydrogels can be self-healed after 2 h, and the healed FFC3 hydrogels can be pulled with forceps without rupturing (see Figure 4g).

### 3.6 Sensing Applications of PPG/ADA-Eu^3+^ Hydrogels

Luminescent lanthanide-containing hydrogels are promising as sensors due to their low cost, high sensitivity, and convenient operations.^14,16,31,32^ In particular, the pH-responsiveness of the lanthanide complexes makes the lanthanide-containing hydrogels become promising pH indicators.^9,33−35^ Here, the pH-sensitive luminescence of the FFC3 hydrogel was demonstrated by adding Tris buffers with different pH values and different volumes to the lyophilized FFC3 hydrogel, and the luminescence of the FFC3 hydrogel was recorded after lyophilization. The results showed that the characteristic luminescent peak (616 nm) of FFC3 hydrogel significantly decreased at the lowest pH value of 5 and the highest volume of 200 μL (see Supporting Information Figure S11).

The pH-sensitive luminescence of the FFC3 hydrogel was further applied in monitoring bacterial growth. Bacterial respiration and fermentation generally produce various volatile acids (e.g., acetic acid, propionic acids, and butyric acids) and base species (e.g., ammonia), depending on the specific metabolic pathways involved.^36−39^ Therefore, the pH value becomes a significant parameter for monitoring bacterial growth and metabolism, as numerous bacteria break down organic materials and generate acids and bases.^40,41^

**Figure 5:**
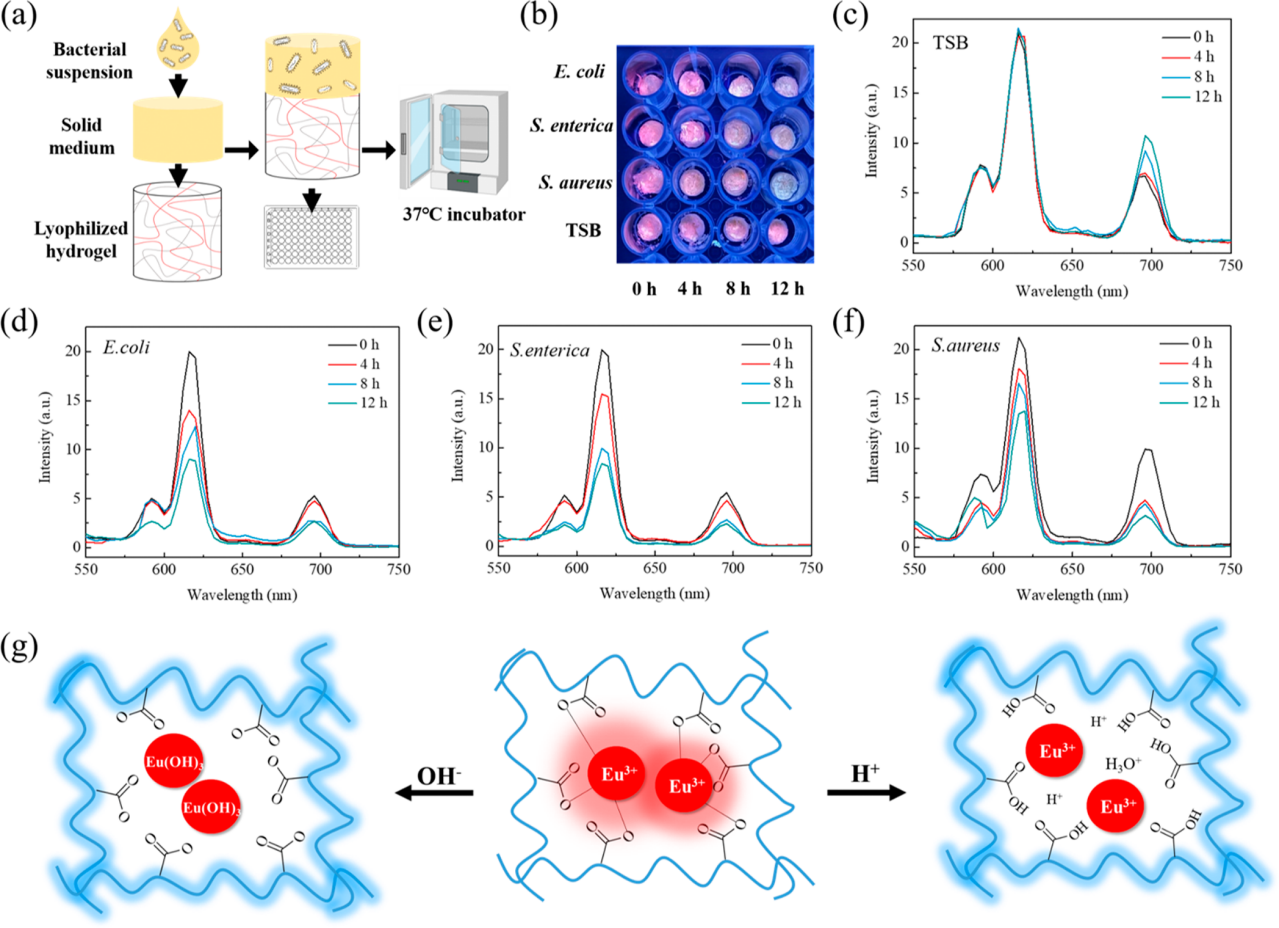
(a) Schematic illustration of the bacterial monitoring using the lyophilized FFC3 hydrogel. (b) Optical images of the lyophilized hydrogels under UV light after the addition of TSB in the absence and presence of bacteria. Luminescence spectra of lyophilized hydrogels after the addition of (c) TSB, (d) *E. coli*, (e) *S. enterica*, and (f) *S. aureus*. (g) Schematic illustration of the effects of pH in the changes of the luminescence property of the hydrogel network.

In our design, agar disks formed by tryptic soy broth (TSB) solid medium were prepared for bacteria culture and placed on top of the lyophilized FFC3 hydrogel (see Figure 5a). TSB in the absence or presence of bacteria (OD ≅ 0.3, 5 μL) was dropped on the solid medium, and the luminescence changes of FFC3 hydrogels were recorded after lyophilization to reduce the influence of water on luminescence quenching. In the control group (i.e., TSB), the luminescence of the lyophilized FFC3 hydrogels at 616 nm rarely declined, indicating that variations in the media composition did not significantly affect the outcomes of bacterial infection assessment (Figure 5b,c). On the other hand, luminescence reductions of 9.5, 24.2, and 23.8% at 616 nm were observed in the FFC3 hydrogels incubated with *E. coli*, *S. enterica*, and *S. aureus*, respectively, after 4 h of incubation (see Figure 5b,d–f). In the end of the experiment, luminescence reductions of 21.5, 59.6, and 54.0% were recorded in FFC3 hydrogels incubated with *E. coli*, *S. enterica*, and *S. aureus*, respectively. The luminescence intensity changes of FFC3 hydrogels observed at 616 nm showed a correlation to the growth of bacteria over time (see Supporting Information Figure S12), indicating the ability of using the lyophilized FFC3 hydrogels to monitor the bacteria growth.

The potential underlying mechanism for the observed luminescence quenching of hydrogels in response to volatile acid and base species can be explained by the complexation changes between Eu^3+^ ions and carboxyl groups^23^ (see Figure 5g). When the environmental pH falls below the constant p*K*_a_ values of alginate (specifically, mannuronic and guluronic acids at 3.38 and 3.65, respectively), the carboxyl groups in ADA are progressively or fully protonated. This leads to a reduction in the strength of complexation between Eu^3+^ ions and the carboxyl groups. Concurrently, the competition between Eu^3+^ ions and H^+^ (or H_3_O^+^) ions increases due to the increased concentration of H^+^. Conversely, as the environmental pH rises, Eu^3+^ hydroxide adducts (Eu(OH)_3_) form, which reduces the activity of Eu^3+^ ions.^42^ To demonstrate the pH influence in the coordination of Eu^3+^ ions in the hydrogel network, the XPS spectra of the FFC3 hydrogel immersed in different pH environments were further investigated. The XPS O 1s spectra of FFC3 hydrogel revealed that the intensity of the Eu–O bond significantly reduced at lower pH values, indicating the binding between the Eu^3+^ ions, and the carboxylate groups of the alginate decreased with increasing acidity of the immersion environment (see Supporting Information Figure S13).

Numerous studies have utilized pH-sensitive hydrogels containing luminescent materials for bacterial sensing applications, where most strategies rely on incorporating pH-sensitive dyes or one/multiple fluorescent molecules into the hydrogel structure.^41,43,44^ Additionally, some studies have employed ion cross-linking to enhance and regulate fluorescence of hydrogels for bacterial detection.^45^ However, few of reports focused on using lanthanide ions as the luminescent source in fabricating hydrogels for bacterial sensing.^40^ Lanthanide complexes with small size can easily hybridize with the hydrogel structure at the molecular level, offering advantages in synthesis. This facilitates the preparation of high-quality luminescent hydrogels with a uniform structure and stable performance for sensing applications. In this study, we demonstrated that the FFC3 hydrogels presented pH-sensitive luminescence as well as showed potential in monitoring bacterial growth. It should be noted that the phenomena responsive to acids and bases using the lanthanide-containing hydrogels are consistent with the reported results.^40,46^ Compared to reported hydrogels with two types of lanthanide ions (i.e., Eu^3+^ and Tb^3+^) in the sensing platform, here, the lanthanide amount in the hydrogels was optimized through the FFC process, allowing for the use of a single type of lanthanide ions (i.e., Eu^3+^) for pH detection and bacterial growth monitoring. Also, the triple-cross-linked network in the PPG/ADA-Eu^3+^ hydrogels can provide a more robust network with good stability for sensing applications in various environments.

In addition to monitoring the growth of bacteria, we also demonstrated that the PPG/ADA-Eu^3+^ hydrogels were capable of sensing copper(II) (Cu^2+^) ions.^32,47−50^ The FFC3 hydrogels were immersed in solutions containing various metal ions (Ca^2+^, Mg^2+^, Ni^2+^, Co^2+^, Cu^2+^, Zn^2+^, and Fe^2+^), and the luminescence changes of FFC3 hydrogels were observed and investigated under UV irradiation. Among the tested metal ions, the Cu^2+^ ions notably reduced the luminescence intensity of FFC3 hydrogels (see Supporting Information Figure S14). The coordination of Cu^2+^ ions with functional groups in the hydrogel network can change the original coordination sphere, increasing the distances between carboxyl and hydroxyl groups and Eu^3+^ ions, resulting in insufficient energy transfer from the functional groups to the Eu^3+^ ions and the subsequent quenching of the red luminescence of hydrogels.

### 3.7 Cytocompatibility of Hydrogels

The cytocompatibility of hydrogels was investigated using the alamarBlue assay, where the hydrogel extract was prepared by immersing the hydrogels into the media for cell culture experiments. However, it was noticed that the extract solutions of FFC1-FFC3 hydrogels were acidic (see Supporting Information Figure S15a), likely due to the presence of NO_3_^2–^ as Eu(NO_3_)_3_ solution (0.01 M, pH = 5.2) was used in the FDS process. Therefore, only the FFC0 hydrogel was cytocompatible, while the FFC1-FFC3 hydrogels were toxic to cells (see Supporting Information Figure S15b). These PPG/ADA-Eu^3+^ hydrogels were more suitable for use in nonbiomedical fields, such as anticounterfeiting materials,^51,52^ optical devices,^53^ data security storage,^54^ and tuning soft actuators.^55,56^ Besides, to prevent the acidity problem, Eu(NO_3_)_3_ solution used in the preparation process of the PPG/ADA-Eu^3+^ hydrogels could be changed to another precursor solution, such as Eu(Cl)_3_, to provide a neutral environment for hydrogels.^57,58^ The neutralization of PPG/ADA-Eu^3+^ hydrogels could also be helpful before using these hydrogels for biomedical applications.

## 4 Conclusions

In this study, we have developed a series of luminescent triple-cross-linked hydrogels utilizing a synergistic method combining freeze-drying-swelling (FDS) and freeze–thawing (FT) processes. The incorporation of Eu^3+^ ions via the FDS process increased the formation of intermolecular coordination cross-links, resulting in mechanically strong and stable hydrogels. Additionally, the FT process contributed to the creation of microcrystalline regions within the hydrogel, further enhancing the mechanical strength of the network. The number of FDS-FT cycles was crucial in defining the microstructures and properties of the hydrogels, showing that the FFC3 hydrogel exhibited the smallest pore size, highest compressive modulus, and lowest swelling ratio among the hydrogel samples. The presence of dynamic bonds in the FFC hydrogels provided them with stimuli-responsive and self-healing capabilities, broadening their potential applications. In particular, contrasting with the commonly used Ca^2+^ ions for cross-linking alginate, Eu^3+^ ions introduced unique luminescent properties to make these hydrogels suitable for sensing applications. Therefore, we also demonstrated the utility of the FFC3 hydrogel in monitoring bacterial growth through the luminescence quenching behavior of the hydrogels induced by the microbial volatile compounds produced by bacterial metabolism. The FFC3 hydrogel can be also used as a metal ion sensor to detect Cu^2+^ ions. Taken together, this research presents a novel approach to fabricating luminescent lanthanide-containing hydrogels with adjustable properties and demonstrates their potential applications in monitoring bacterial growth and detecting Cu^2+^ ions.

## Supporting Information Available

^1^H NMR and FT-IR spectra of alginate, ADA, PEI–PBA, gelatin, and PPG; GPC profiles of alginate, ADA, gelatin, and PPG; full-range XPS spectra of hydrogels; MIP data, SEM elemental distribution analysis, and stress–strain curves of hydrogels; compressive modulus of FT hydrogels; photos and curves of the FFC3 hydrogels under repetitive compression loading; optical images and luminescence spectra of the hydrogels at different acidic conditions; changes in hydrogel luminescence throughout bacterial growth; O 1s XPS spectra of FFC3 hydrogels at different acidic conditions; luminescence responses of the FFC3 hydrogels soaked in different metal ion solutions; normalized metabolic activity of cells cultured in the extract solutions of hydrogels (PDF)
