# Supplementary material for: Fabrication of Luminescent Triple-Cross-Linked Gelatin/Alginate Hydrogels through Freezing-Drying-Swelling and Freezing-Thawing Processes
Source: Biomacromolecules. 2024 Aug 15;25(9):5758–70. doi: 10.1021/acs.biomac.4c00289 (PMC11388451; doi:10.1021/acs.biomac.4c00289)
Supplement: Supplementary file 1 — bm4c00289_si_001.pdf [file bm4c00289_si_001.pdf]

Supporting information

Fabrication of Luminescent Triple-Cross-Linked Gelatin/Alginate Hydrogels through Freezing-Drying-Swelling and Freezing-Thawing Processes

Ting-Hsiang Chiu[a], Shu-Ying Wu[a], Yi-Chen Yang[a], Chen-Jie Yan[a], and Yi-Cheun Yeh[a*]

[a] Institute of Polymer Science and Engineering, National Taiwan University, Taipei, Taiwan.

* Corresponding author.

E-mail address: yicheun@ntu.edu.tw (Y.-C. Yeh)

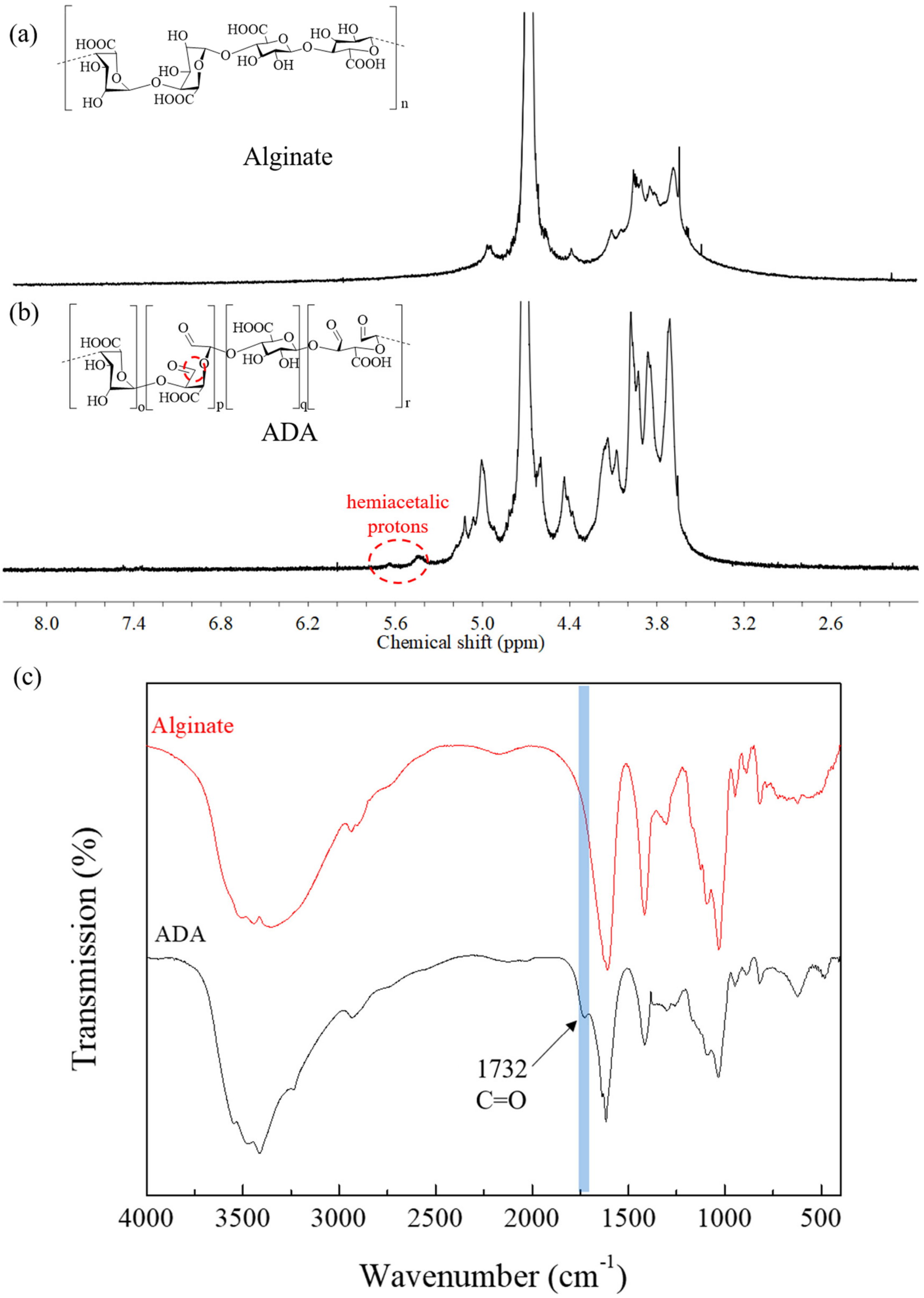

Figure S1. (a-b) ^{1}H-NMR and (c) FT-IR spectra of alginate and ADA.

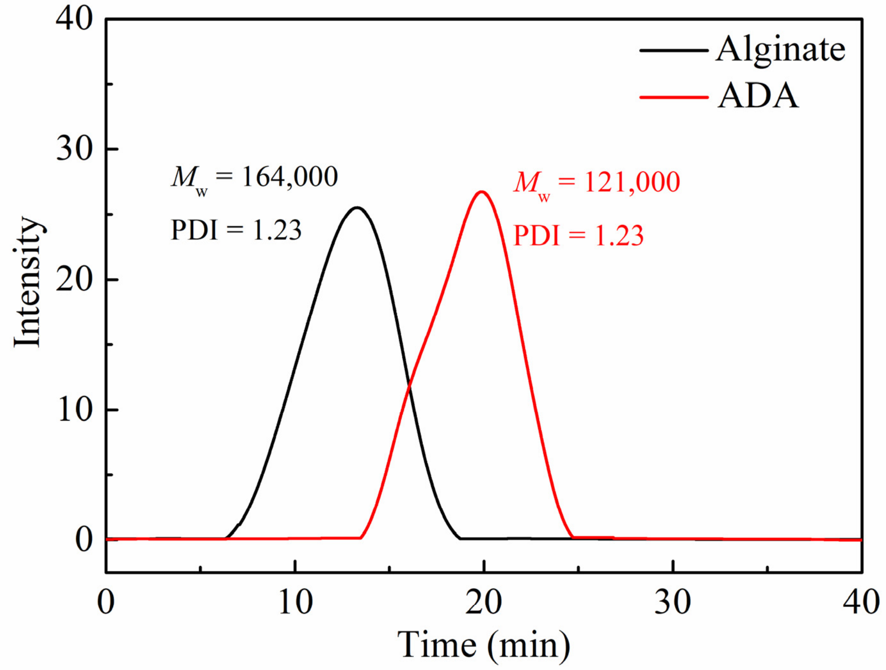

Figure S2. GPC profiles of alginate and ADA.

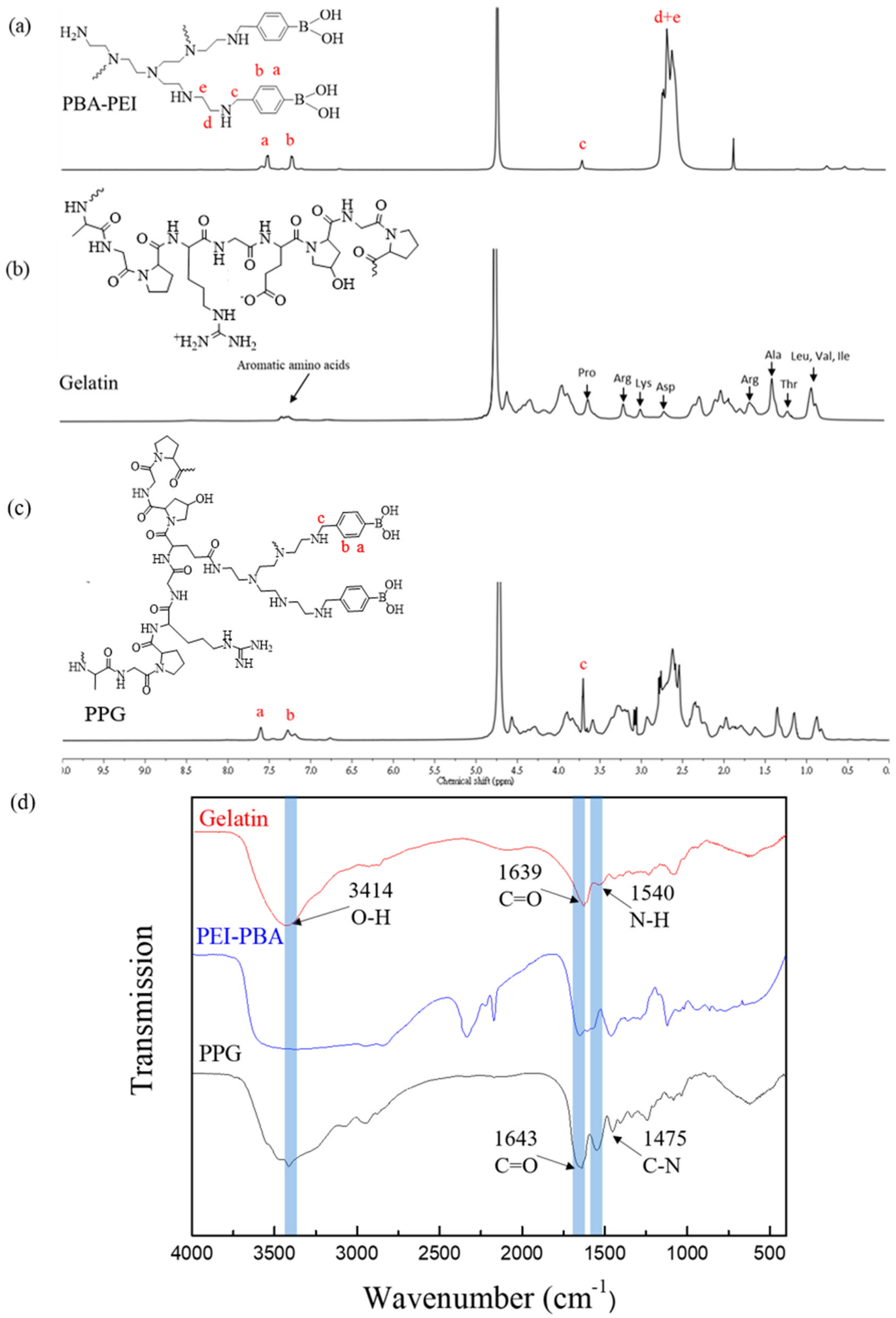

Figure S3. (a-c) ^{1}H NMR and (d) FT-IR spectra of PEI-PBA, gelatin, and PPG.

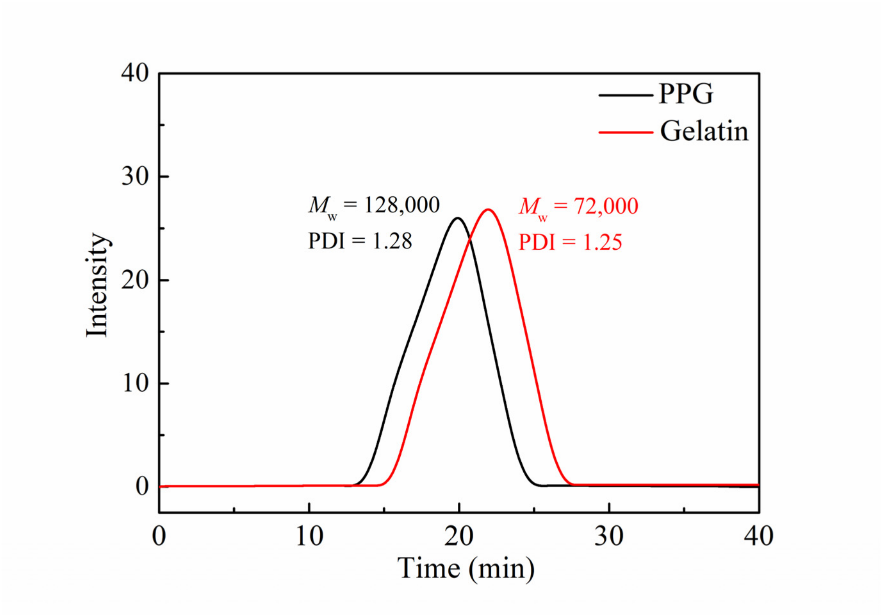

Figure S4. GPC profiles of gelatin and PPG.

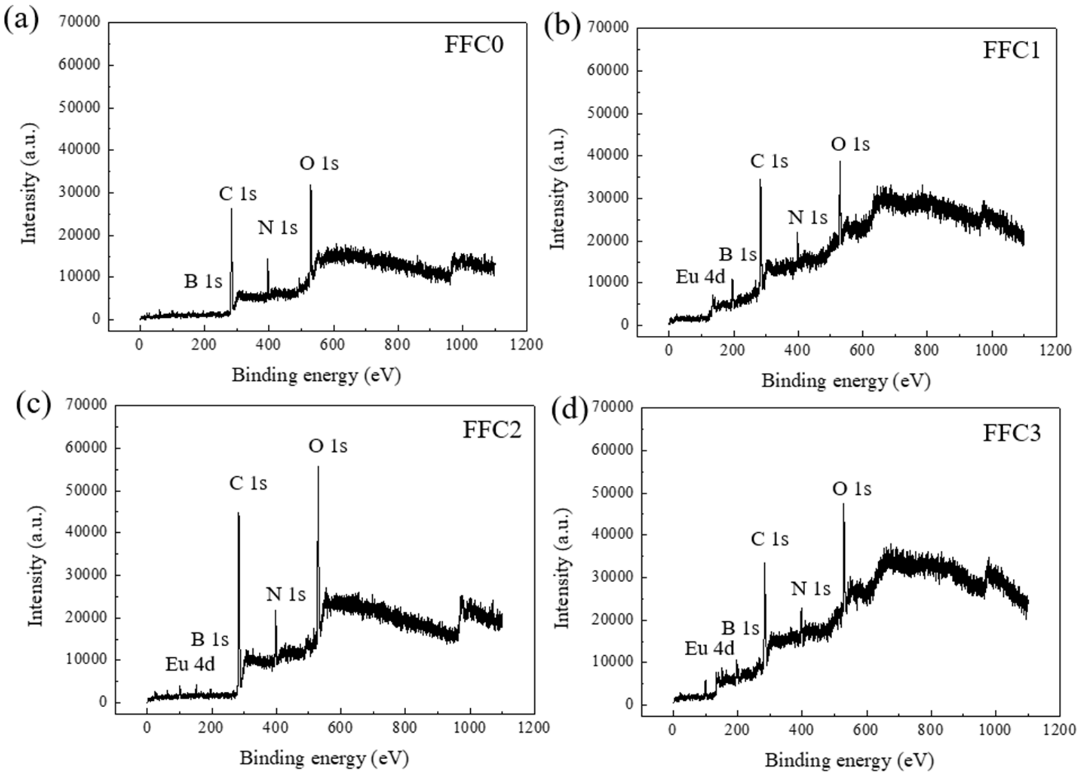

Figure S5. Full-range XPS spectra of hydrogels.

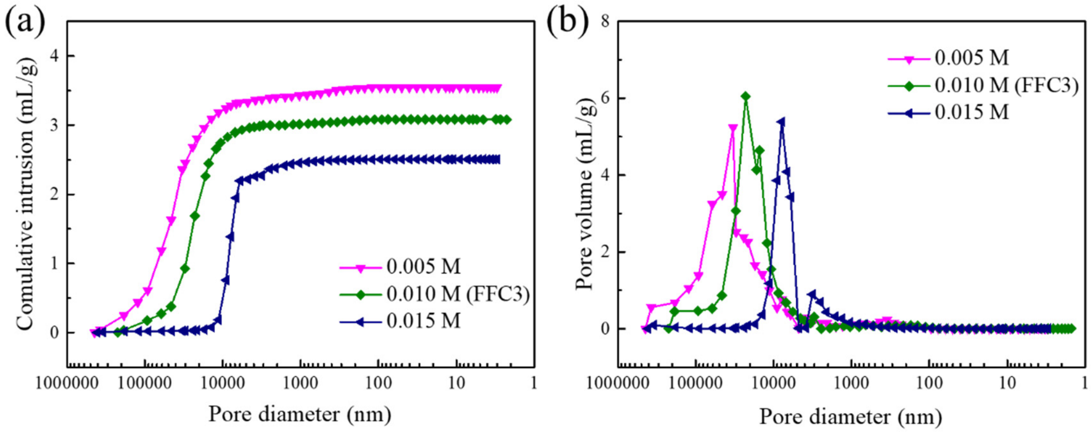

Figure S6. (a) Cumulative pore volume and (b) differential pore volume (dV/dD) of hydrogels prepared by immersing the hydrogels with different concentrations of $Eu(NO_3)_3$ solutions (i.e., 0.005, 0.010 (FFC3), and 0.015 M).

Table S1. MIP analyses of hydrogels.

Hydrogels prepared with different Eu^{3+} concentration	Total intrusion volume (mL/g)	Median pore diameter (μm)	Total pore area (m^2/g)	Porosity (%)
0.005 M	3.55	42.50	1.90	85.13
0.010 M (FFC3)	3.08	24.34	1.68	86.24
0.015 M	2.51	8.11	2.04	87.39

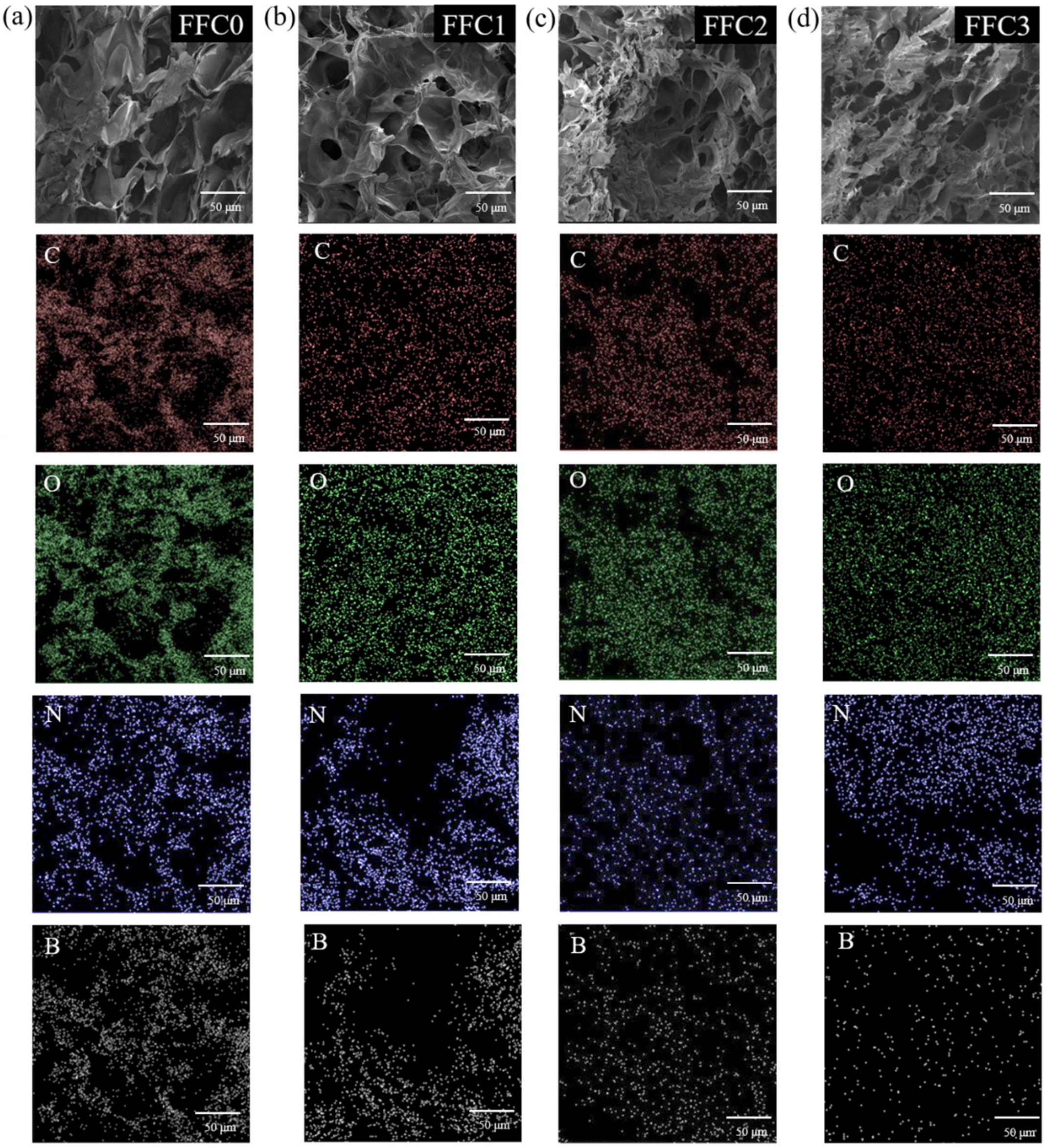

Figure S7. SEM elemental distribution analysis of hydrogels, showing the distribution of carbon/oxygen/nitrogen/boron elements.

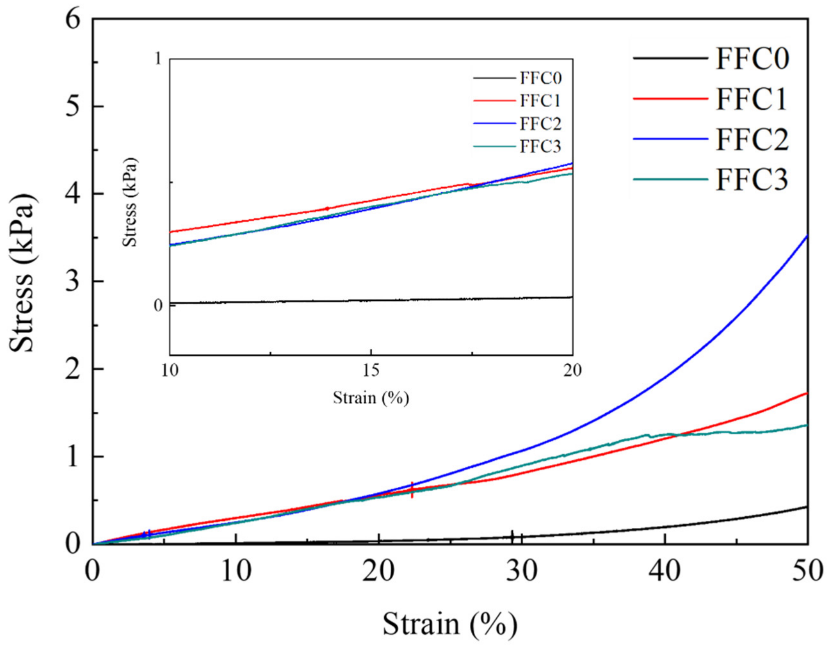

Figure S8. Representative stress-strain curves of FFC hydrogels.

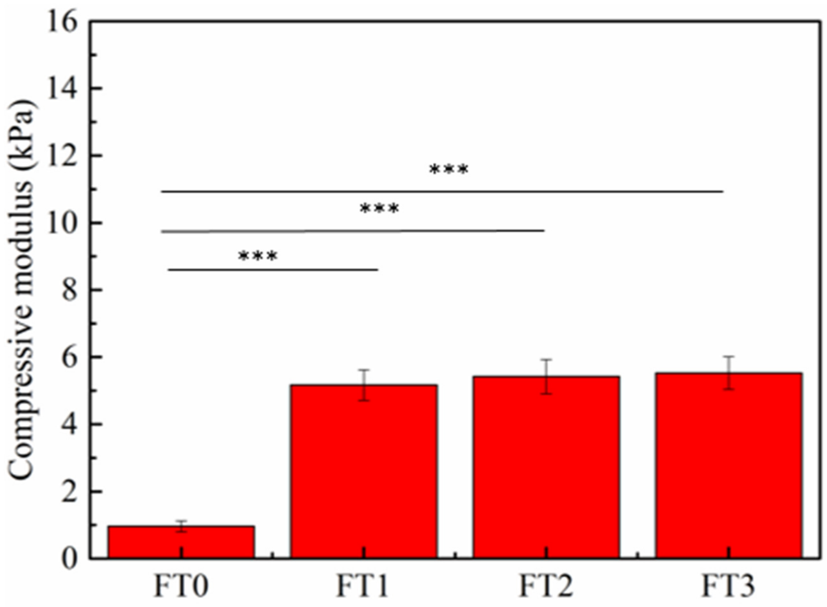

Figure S9. Compressive modulus of FT hydrogels. The significance was set as $p < 0.05$ with *** indicating $p < 0.001$, respectively.

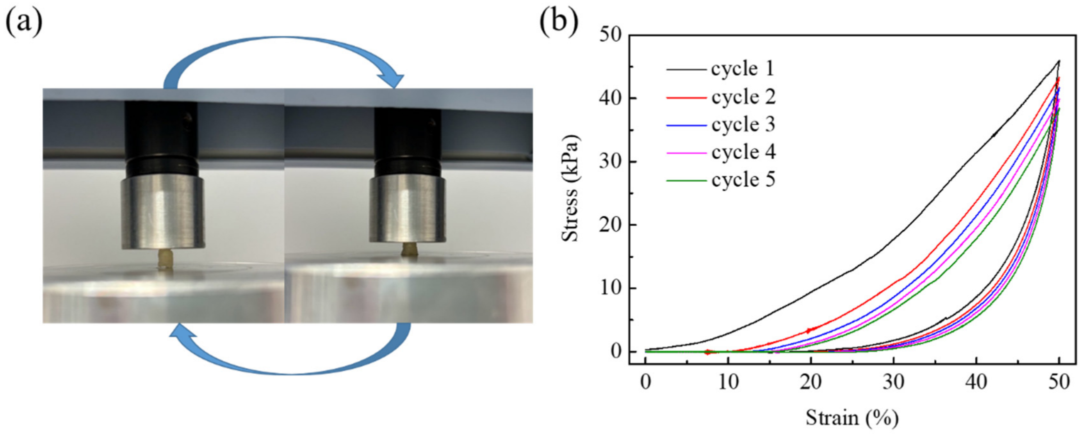

Figure S10. (a) Photos and (b) curves of the FFC3 hydrogels under repetitive compression loading to 50% strain (five cycles at a crosshead speed of 10 mm/min).

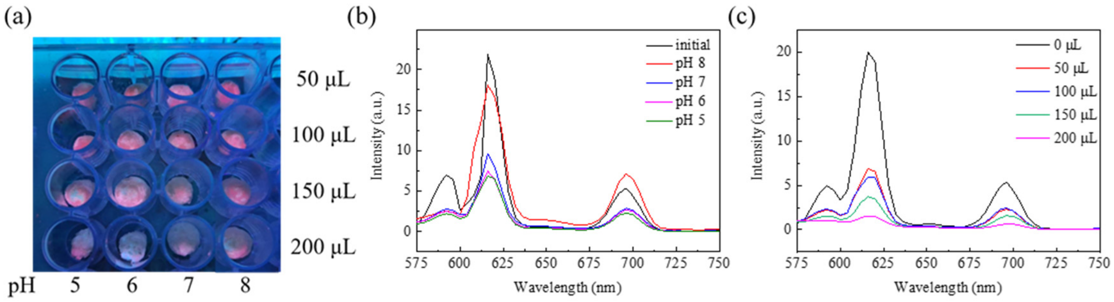

Figure S11. (a) Optical images of the hydrogels (diameter= 7 mm, height= 6 mm, prepared through the volume of 300 μL) under UV after adding Tris buffer with different pH values and volumes. (b) Luminescence spectra of hydrogels after adding Tris buffer (50 μL) with different pH values. (c) Luminescence spectra of hydrogels after adding Tris buffer (pH 5) with different volumes.

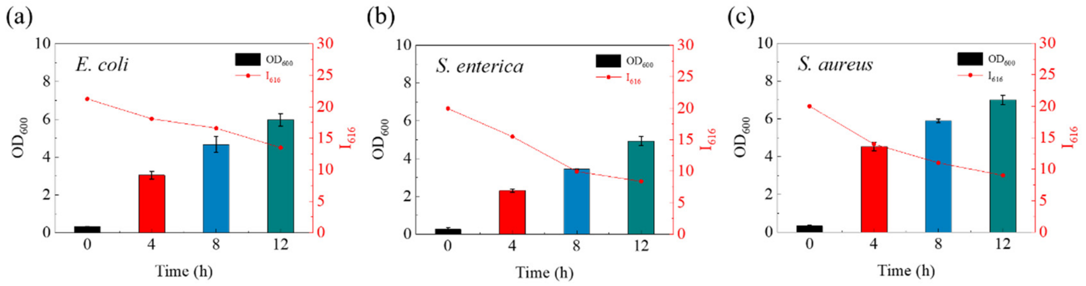

Figure S12. The changes in hydrogel luminescence throughout bacterial growth: (a) *E. coli*, (b) *S. enterica*, and (c) *S. aureus*.

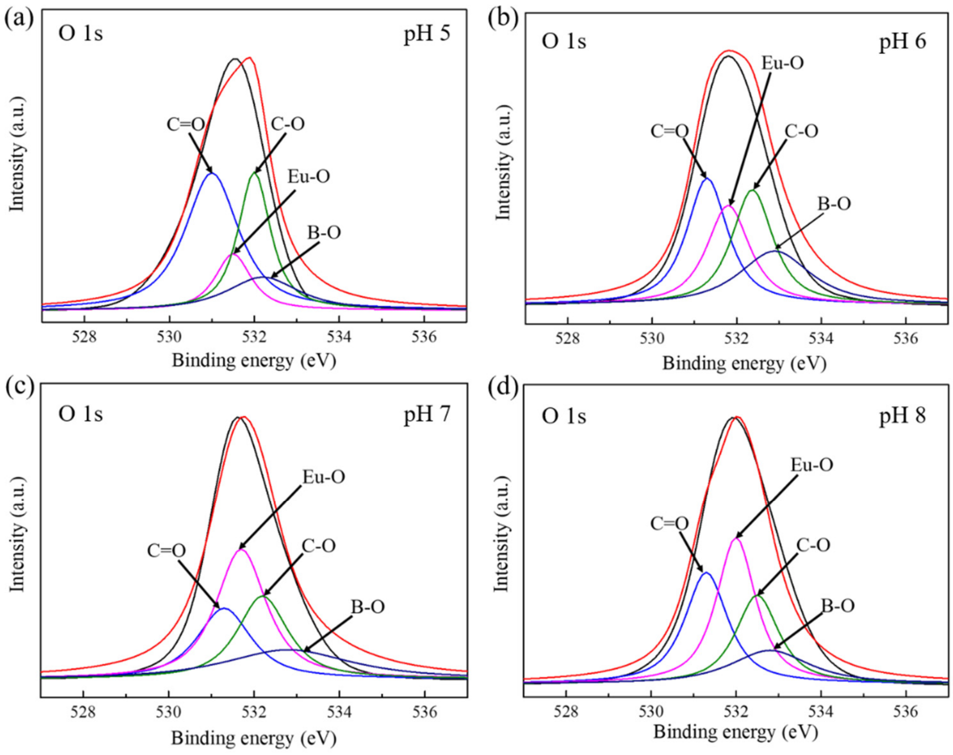

Figure S13. O1s XPS spectra of FFC3 hydrogels: (a) pH 5, (b) pH 6, (c) pH 7, and (d) pH 8. The FFC3 hydrogels (0.1 ml) were prepared by soaking in various pH solutions (1 mL) for 6 hrs, and then lyophilized using a freeze dryer overnight.

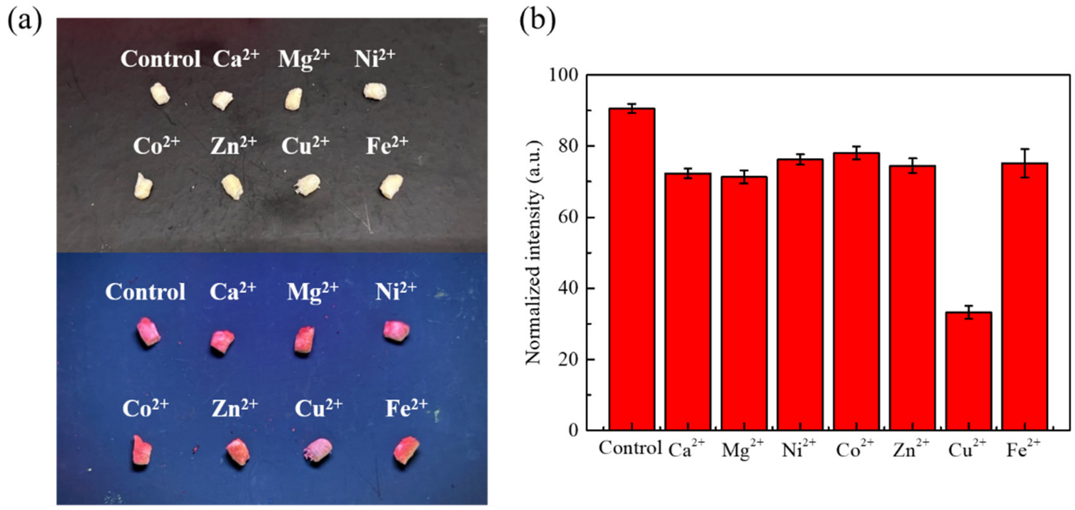

Figure S14. (a) Photos of the FFC3 hydrogels under visible light and UV irradiation (365 nm) after immersing in varios metal ion solutions. (b) Luminescence responses of the FFC3 hydrogels soaked in different metal ion solutions.

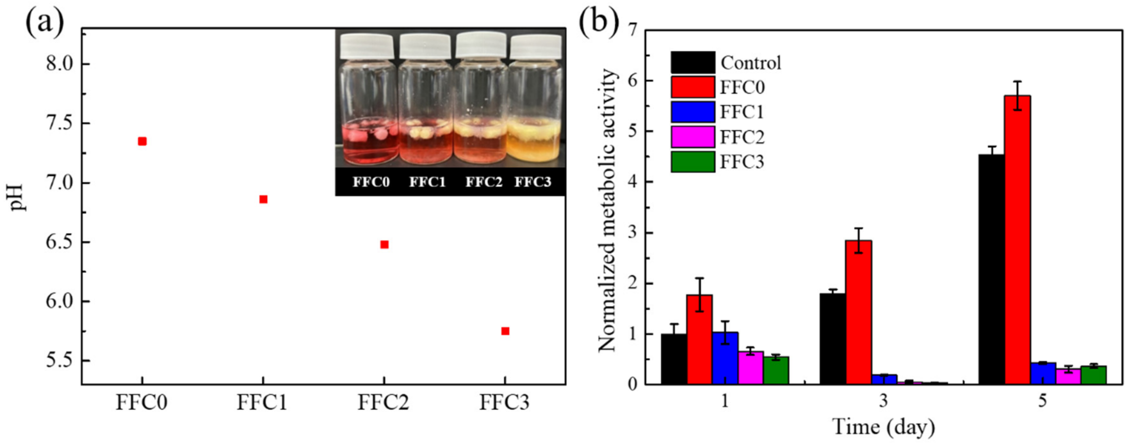

Figure S15. (a) Photos of the hydrogels immersed in media, and the pH values of the extract solutions of hydrogels. (b) Normalized metabolic activity of MEFs cultured in the extract solutions of hydrogels.
